# Outcomes of Peptide Vaccine GV1001 Treatment in a Murine Model of Acute Noise-Induced Hearing Loss

**DOI:** 10.3390/antiox9020112

**Published:** 2020-01-27

**Authors:** Sang-Yeon Lee, Jae Joon Han, Sang-Youp Lee, Gaon Jung, Hyun Jin Min, Jae-Jin Song, Ja-Won Koo

**Affiliations:** 1 Department of Otorhinolaryngology-Head and Neck Surgery, Seoul National University Bundang Hospital, Seoul National University College of Medicine, Seongnam 463-707, Korea; maru4843@hanmail.net (S.-Y.L.); gaon0313@naver.com (G.J.); mhj830108@naver.com (H.J.M.); jjsong96@gmail.com (J.-J.S.); 2 Department of Otorhinolaryngology-Head and Neck Surgery, Soonchunhyang University College of Medicine, Seoul Hospital, Seoul 04401, Korea; seagulla@naver.com; 3 Department of Otolaryngology, Wonkwang University Hospital, Wonkwang University School of Medicine, Iksan 15865, Korea; lsy738@hanmail.net

**Keywords:** noise-induced hearing loss, GV1001, oxidative stress

## Abstract

Noise-induced hearing loss (NIHL) is primarily caused by damage to cochlear hair cells, associated with synaptopathy. The novel cell-penetrating peptide GV1001, an antitumor agent, also has antioxidant and anti-inflammatory effects, and is otoprotective in a murine model of kanamycin-induced ototoxicity. Here, we explored whether GV1001 attenuated NIHL, and the underlying mechanism at play. We established an NIHL model by exposing 4- to 6-week-old C57/BL6 mice to white noise at 120 dB SPL for 2 h, resulting in a significant permanent threshold shift (PTS). We then subcutaneously injected saline (control), GV1001, or dexamethasone immediately after cessation of PTS-noise exposure and evaluated the threshold shifts, structural damages to outer hair cells (OHCs), and ribbon synapses. We also verified whether GV1001 attenuates oxidative stress at the level of lipid peroxidation or protein nitration in OHCs 1 h after exposure to white noise at 120 dB SPL. GV1001-treated mice exhibited significantly less hearing threshold shifts over 2 weeks and preserved OHCs and ribbon synapses compared with controls. Similarly, dexamethasone-treated mice showed comparable protection against NIHL. Importantly, GV1001 markedly attenuated oxidative stress in OHCs. Our findings suggest that GV1001 may protect against NIHL by lowering oxidative stress and may serve as preventive or adjuvant treatment.

## 1. Introduction

Overexposure to intense sound, one of the most common occupational hazards, is a major cause of sensorineural hearing loss (SNHL). Noise-induced hearing loss (NIHL) is mainly caused by damage to hair cells in the cochlea and is associated with synaptopathy [1,2]. Overexposure to intense sound, even for a short time period, can trigger temporary or permanent threshold shift (PTS), depending on the extent of imbalance between oxidative stress mediating cell stress and intrinsic cellular defense mechanism including autophagy [3]. NIHL reportedly elicited progressive consequences: the neural response declines in amplitude, hearing threshold is enhanced, and hair cell numbers fall [4]. Although the exact mechanism remains elusive, overexpression of free radicals, including reactive oxygen species (ROS) and reactive nitrogen species (RNS), followed by lipid peroxidation and protein nitrogen, develop a positive feedback loop for vasoconstriction, resulting in oxidative damage to the cochlea during noise exposure [5]. Currently, several damage mediators relevant to NIHL, such as free radical, intracellular free Ca^2+^, inflammatory mediators, and cell stress or apoptosis-related genes, and their signaling cascades, have been suggested as potential cellular mechanisms to cause cell death in the hearing organ [6]. Although several issues limit the translation of potential protectants based on cellular mechanisms to prevent noise damage, the development of new therapeutic strategies against oxidative stress-mediated conditions may confer the additional basis for the understanding of mechanisms and the translational potential protection on NIHL.

GV1001, a novel cell-penetrating peptide (16-amino-acid sequence) derived from human telomerase reverse transcriptase, has been implicated to have anticancer, antioxidant, anti-inflammatory, and antiapoptotic effects in kidney; it prevents myocardial ischemia-reperfusion injury; stimulates neural stem cell; and is active against various cancer types [7,8,9,10,11]. Moreover, our previous studies revealed that GV1001 significantly attenuated hearing threshold shift and cochlear outer hair cell (OHC) damage in kanamycin-induced ototoxicity mouse model [12,13]. Given that antibiotics-induced ototoxicity has been associated with oxidative stress as a potential mechanism for damage [14], GV1001 may effectively attenuate kanamycin-induced ototoxicity toward sensory hair cells by downregulating ROS/RNS overproduction and their subsequent byproducts. Furthermore, pleiotropic properties of GV1001 may offer additional protective mechanisms against ototoxicity by blocking the upregulation of pro-inflammatory mediators and proapoptotic caspases induced by kanamycin. Taking into consideration previous studies regarding the therapeutic effects of GV1001, we hypothesized that GV1001 might also exert otoprotective effects against NIHL, because excessive ROS/RNS generation and its signaling cascades, involving inflammatory and cell stress or apoptosis pathways are thought to underlie the causative mechanisms of NIHL. In support of this hypothesis, Avenanthramide-C that possesses antioxidant and anti-inflammatory properties was shown to exhibit a similar protective effect against noise- and drug-induced ototoxicity in the mouse model [15]. To the best of our knowledge, there have been no studies regarding the therapeutic effects of peptide vaccine GV1001 on NIHL.

To test our hypothesis, we first developed the noise exposure protocol that caused PTS in 4–6-week-old female C57/BL6 mice. We then evaluated serial hearing threshold shifts and hair cell damage to investigate whether GV1001 exerts the otoprotective effects against NIHL. Previous animal studies have shown that the use of steroids, such as dexamethasone, exert reliable otoprotective effects against inner ear damage caused by noise overexposure [16,17]. Moreover, a recent human study demonstrated the ameliorating effect of dexamethasone on acoustic trauma [18]. Thus, the current study also compared the otoprotective effects of GV1001 and dexamethasone in terms of the extent of threshold shifts and cochlear hair cell and synapse damage. Additionally, we focused on the levels of oxidative stress, as indicated by-products of lipid peroxidation and protein nitration, to elucidate the mechanism of GV1001 effect on NIHL.

## 2. Materials and Methods

### 2.1. Study Design

Female 4–6-week-old C57/BL6 mice (weight, 15–25 g) were used and were acclimatized under specific pathogen-free conditions with a 12 h light/dark cycle for 1 week. We first characterized noise exposure strategies to generate an appropriate PTS mouse model. Mice were exposed in an electrically shielded, double-walled soundproof chamber. The noise stimulus, white noise with a frequency spectrum from 1 kHz to 20 kHz, was presented through dynamic loudspeakers (JBL 2446H/J) positioned at the top of the apparatus. The sound was verified by sound level meter (Bruel & Kjaer Type 2250 s/n2473347) and sound calibrator (Bruel & Kjaer Type 4231 s/n1883578). 

**Figure 1:**
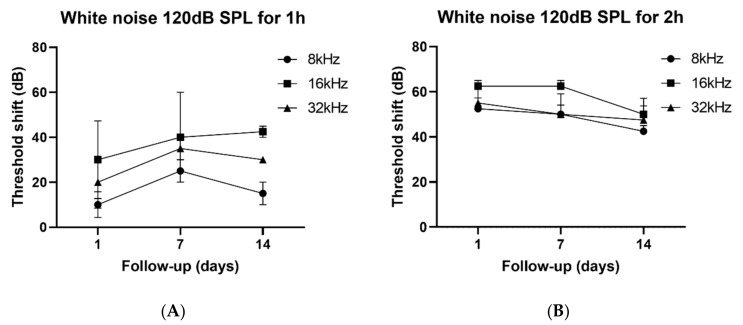
Noise exposure strategies: white noise 120 dB SPL for 1 h ((**A**) moderate permanent threshold shift (PTS), *n* = 4) and 2 h ((**B**) severe PTS, *n* = 4). The baseline auditory brainstem response (ABR) thresholds for moderate PTS (dB SPL) were 30 ± 4.08 at 8 kHz), 30 ± 5.77 at 16 kHz, and 35 ± 2.89 dB at 32 kHz, respectively. The baseline ABR thresholds for severe PTS (dB SPL) were 35 ± 2.89 at 8 kHz, 32.5 ± 2.50 at 16 kHz, and 40 ± 4.08 at 32 kHz, respectively. The Error bar indicates SEM.

Exposure to white noise at 120 dB SPL for 1 h resulted in a moderate threshold shift of 10, 30, and 20 dB at 8, 16, and 32 kHz, respectively, on day 1 after noise exposure (*n* = 4, 8 ears). The hearing threshold remained stable or slightly increased over 2 weeks throughout all frequencies, indicating that the noise exposure strategy had elicited moderate PTS (Figure 1A). On the other hand, exposure to white noise at 120 dB SPL for 2 h resulted in significant threshold shifts of 60, 70, and 70 dB at 8, 16, and 32 kHz, respectively, which remained constant over 2 weeks, despite partial attenuation of hearing threshold over time (*n* = 4, 8 ears). The exposure to white noise at 120 dB SPL for 2 h had elicited severe PTS (Figure 1B). The study protocols were approved by the Institutional Animal Care and Use Committee of Seoul National University Bundang Hospital (No. 52-2018-001). Date of approval was 21 March 2019.

We used the severe PTS-noise protocol to explore whether GV1001 was otoprotective against NIHL in terms of the hearing threshold shift and hair cell morphology. Mice were allocated to three groups: saline (control, *n* = 17), GV1001 (10 mg/kg; GemVax & Kael Co., Ltd., Seongnam, Korea, *n* = 17), and dexamethasone (15 mg/kg, *n* = 17); each mouse received the drug once via a subcutaneous route immediately after cessation of severe PTS-noise. Each injection material was injected with 0.1 mL per 20 g of mouse. Auditory brainstem response (ABR) thresholds were measured immediately and 1, 7, and 14 days later. Damage to OHCs and ribbon synapses were evaluated in whole-mounting section 14 days after noise exposure. As for experiment on ribbon synapses counting, an additional four mice per group were used for evaluating the effect of GV1001, dexamethasone, and saline on ribbon synapses after severe PTS-noise.

An additional experiment was conducted to investigate whether GV1001 attenuates the levels of oxidative stress. Before noise exposure, each material was injected once via the subcutaneous route, as described above. Based on a previous study [3], markers of oxidative stress, lipid peroxidation (4-HNE) and protein nitration (3-NT), were evaluated in OHCs on surface preparations at 1 h after exposure to white noise at 120 dB SPL (moderate PTS). Each group (GV1001 group and saline group) contained 5 mice for an additional experiment. Collectively, a total of 69 mice were used for this study.

### 2.2. ABR Threshold Recording

As described previously [12,13], we measured ABR thresholds using tone bursts (envelope, Blackman; duration, 1562 ms; stimulation rate, 21.1/s) delivered at 8, 16, and 32 kHz (SmartEP; Intelligent Hearing System; Miami, FL, USA) to the external auditory meatus via plastic earphones connected to an EC1 electrostatic speaker. ABR stimulus was presented in a closed field setting and both the ears tested separately. Subdermal needle electrodes were applied to the vertex and behind the ipsilateral pinna. A subdermal needle electrode behind the contralateral pinna served as a ground electrode. Bandpass filters (100–3000 Hz) were used and data from 1024 sweeps averaged in real-time. Using the decreasing method, the ABR thresholds at each frequency were the lowest sound intensities of Wave III at the point where the most robust and stable component was evoked (around 4 ms). In this study, the sound intensity of the tone burst stimuli was lowered at 10 dB SPL intervals from 90 dB SPL to acquire auditory thresholds. The auditory threshold was defined as the lowest sound intensity at which a visible auditory brainstem response wave was observed. If the hearing threshold could not be measured at 90 dB SPL, the result was reported as threshold of 100 dB SPL.

### 2.3. Tissue Preparation

The method used to determine the proportion of intact OHCs has previously been described [12,13]. Briefly, after mounting, immunolabeled samples were imaged using a Zeiss 710 confocal microscope at a uniform magnification of 63×. In the first experiment, a rabbit polyclonal antibody targeting MYO VIIa (green) (concentration 1:100; Abcam, Inc., Cambridge, MA, USA; ab3481) was used to immunolabel hair cells in surface preparations, enabling identification of comparable OHC regions in confocal images. We first manually counted the numbers of morphologically intact OHCs in five different fields, and then averaged these per cochlear turn. Then we subjected fluorescence signals to semi-quantitative analyses; cells immunolabeled with CtBP-2 (inner hair cells; IHCs) were quantified using ImageJ software (National Institutes of Health, Bethesda, MD, USA) under identical conditions (thus using the parameters of the original confocal images). 

In the second experiment, we immunolabeled the OHCs with a polyclonal rabbit anti-nitrotyrosine antibody (1:50; EMD Millipore; Billerica, MA, USA) and a polyclonal rabbit anti-4-HNE antibody (1:50; Abcam, Inc.; ab46545); both antibodies were tagged with Alexa 488-conjugated phalloidin at a concentration of 1:100 (green). We immunolabeled 4-HNE and 3-NT in six slices selected at random from five mice and quantified OHC staining by reference to the original confocal images; we used ImageJ software to this end.

### 2.4. Statistical analysis

All statistical analyses were performed using R Software (R ver. 3.5.2 and R Studio ver. 1.0.136, Foundation for Statistical Computing, Vienna, Austria) and the results were visualized using Prism Graphpad. All data are shown as means ± standard errors of the means (SEMs). One-way ANOVA and the post hoc Tukey tests were used to compare differences among groups when the data followed a normal distribution. Alternatively, the Kruskal–Wallis test and the post hoc Mann–Whitney U-test were employed when the data were not normally distributed. *p*-values < 0.05 were considered to indicate statistically significant differences. 

## 3. Results

### 3.1. GV1001 Rescues Hearing Threshold Shifts After Severe PTS-Noise

**Figure 2:**
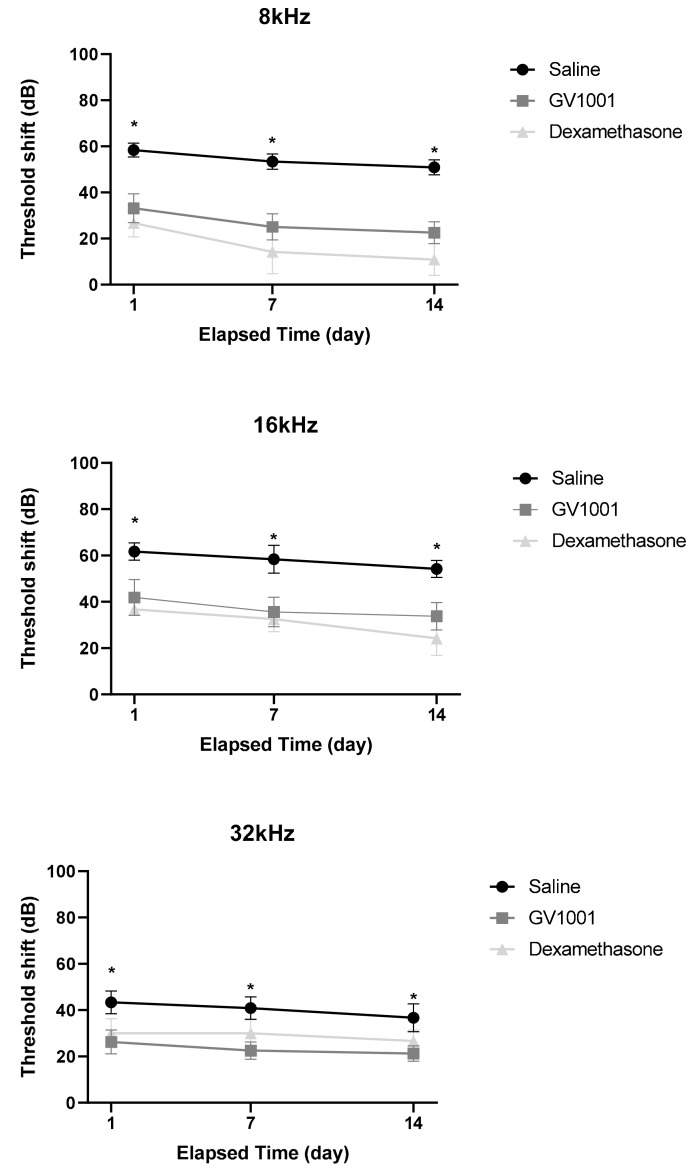
Effect of GV1001, dexamethasone and saline treatment in noise-induced hearing loss (NIHL) murine model induced by severe PTS-noise. Threshold shift (dB) relative to baseline at post-treatment 1 day, 7 days, and 14 days among the GV1001, dexamethasone and saline groups is illustrated according to frequencies. All groups contained 17 mice. The Error bars in threshold shift panels are SEM. * *p* < 0.05 (by one-way ANOVA with the Tukey post-hoc test).

The average post-experimental ABR thresholds at 8, 16, and 32 kHz over 2 weeks among the three groups are shown in Figure 2. Immediately after noise exposure, the average hearing thresholds at 8, 16, and 32 kHz did not differ among the groups. On day 1 after severe PTS-noise, the hearing threshold at 8, 16, and 32 kHz significantly increased in saline-treated mice (8 kHz: 95.0 ± 2.2, 16 kHz: 93.3 ± 2.1, 32 kHz: 93.3 ± 2.1). At day 7, there was a gradual improvement in the hearing threshold, approximately less than 5 dB at all frequencies. On day 14, more improvement was observed. Overall, the hearing threshold at 8, 16, and 32 kHz was 91.7 ± 3.1, 91.7 ± 1.7, and 93.3 ± 3.3, respectively, at the last evaluation (day 14).

At day 1 after severe PTS-noise exposure, compared to saline-treated (control) mice, GV1001-treated mice exhibited significantly lower hearing threshold shifts at 8, 16, and 32 kHz (8 kHz, 67.5 ± 9.1 vs. 95.0 ± 2.2, *p* = 0.006; 16 kHz, 70.6 ± 3.8 vs. 93.3 ± 2.1, *p* = 0.027; 32 kHz, 74.2 ± 5.0 vs. 93.3 ± 2.1, *p* = 0.032). Importantly, differences in the threshold shift between saline control and GV1001 group were 25.21 ± 7.76 dB at 8 kHz, 19.79 ± 9.56 dB at 16 kHz, and 17.08 ± 7.43 dB at 32 kHz, respectively. At days 7 and 14, as in control mice, mild recoveries (<5 dB) of all three hearing thresholds were evident in GV1001-treated mice. Compared to controls, GV1001-treated mice exhibited significantly lower hearing threshold shifts at 8, 16, and 32 kHz. Dexamethasone afforded similar protective effects. The significant differences in the ABR thresholds at 8, 16, and 32 kHz between the dexamethasone and saline groups were maintained over the 2 weeks. The three thresholds did not differ between the GV1001 and dexamethasone groups at any time.

Thus, GV1001 rescued the hearing thresholds at 8, 16, and 32 kHz by approximately 20–30 dB compared to those of NIHL induced by severe PTS-noise. The protective effects of GV1001 were somewhat greater at 8 than 16 and 32 kHz, but statistical significance was not attained. The hearing protection afforded by GV1001 was similar to that imparted by dexamethasone

### 3.2. GV1001 Attenuates Hair Cell Damages Including Synaptic Ribbons

**Figure 3:**
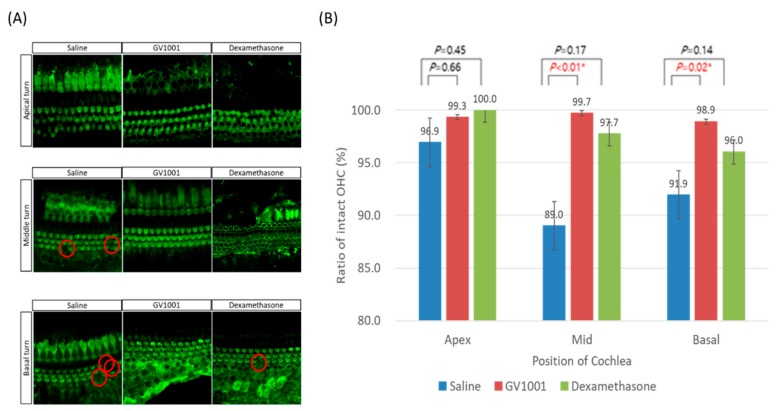
Effect of GV1001, dexamethasone and saline treatment in NIHL murine model induced by severe permanent threshold shift (PTS)-noise. (**A**) Representative images of myosin-VII-labeled hair cells in each cochlear turn on day 14 after severe PTS-noise exposure. Red circles indicate lost outer hair cells (OHCs). Scale bar: 10 µm. (**B**) Survival rate of the OHCs according to different systemic injection after cessation of severe PTS-noise. The Error bars in threshold shift panels are SEM. All groups contained 17 mice.* *p* < 0.05 (by one-way ANOVA with the Tukey post-hoc test).

Upon cochlear whole-mount examination (Figure 3), a variable amount of OHCs were missing or disorganized in saline-treated mice after severe PTS-noise exposure, especially in middle and basal turn. By contrast, GV1001-treated mice showed well-organized OHCs, regardless of cochlear turns. Furthermore, the OHC survival rate was significantly higher in GV1001- than saline-treated mice, particularly in the middle and basal turns. The proportion of intact OHCs was seemingly higher in dexamethasone-treated mice than in saline-treated mice, although it was not statistically significant.

**Figure 4:**
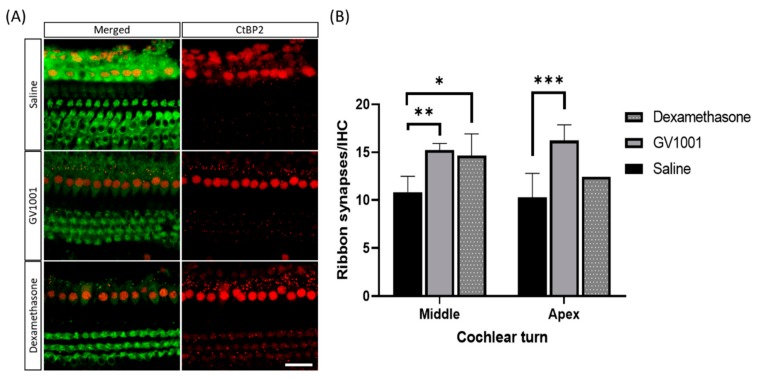
Effect of GV1001, dexamethasone, and saline on ribbon synapses. (**A**) Representative images of CtBP2-labeled ribbon synapses in cochlear apical turns 14 days after severe PTS-noise exposure. Scale bar: 10 µm. Representative images were taken from the middle turn. (**B**) Error bars indicate the SEMs. Significant differences are evident between the saline, and GV1001 and dexamethasone groups. All groups contained 4 mice (*n* = 8 ears). *p < 0.05, **p < 0.005, ***p < 0.001 (by the Kruskal–Wallis test and Mann–Whitney U-test). Scale bar, 10 µm.

Additionally, we manually counted the number of IHC and immunolabeling of CtBP2 on surface preparation from original confocal images, each taken with a 63× magnification lens under identical z-stack conditions. A total of 84 IHCs in 8 sections in saline group, 114 IHCs in 10 sections in GV group, and 30 IHCs in 4 sections in dexamethasone group were identified. Then, the ratios of CtBP2 to IHC were calculated. In this study, CtBP2 puncta per inner hair cell were based upon the total quantitation of middle and apical turns, due to unreliable sectioning in the basal turn of the inner hair cell. Compared with that in the saline-treated mice, CtBP2 was significantly higher in both GV1001- and dexamethasone-treated mice (Figure 4).

### 3.3. GV1001 Decreases the Expression of Oxidative Stress Markers

**Figure 5:**
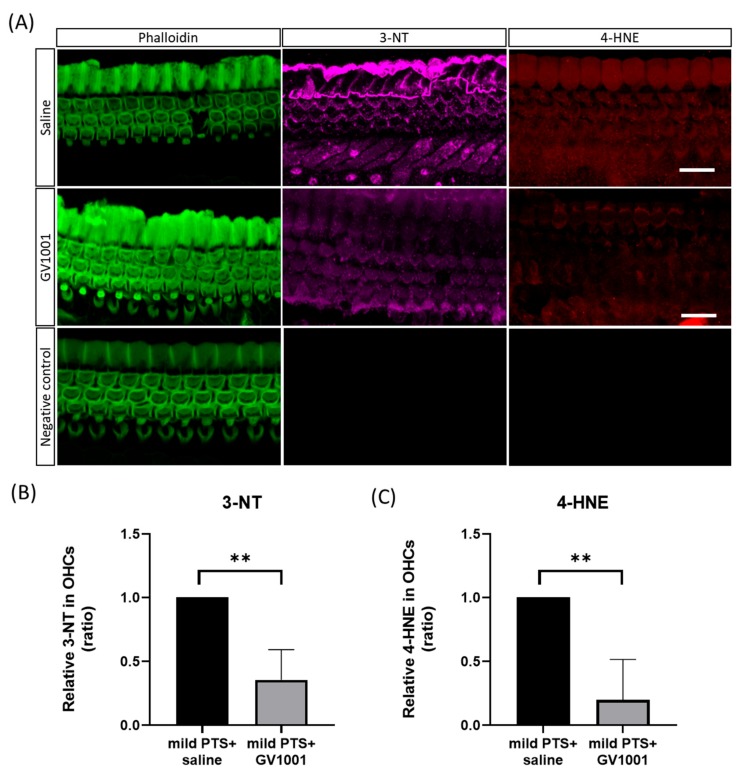
Effect of GV1001 on oxidative stress marker levels. (**A**) Immunolabeled 3-NT (purple) and 4-HNE (red) OHCs stained green with phalloidin 1 h after moderate PTS-noise exposure. The staining intensity of the GV1001 group is markedly weaker than that of the control group. As for negative control, no immunoreactivity was observed in the state of attaching only secondary antibody to verify whether the expression of 3-NT and HNE is reactive by oxidative stress. (**B**,**C**) GV1001 pretreatment significantly attenuated the immunoreactivities of 4-HNE and 3-NT (*n* = 5, 10 ears). All groups contained 5 mice. **p < 0.005 (Mann–Whitney U-test). Scale bar: 10 µm.

We examined the levels of oxidative stress markers (products of lipid peroxidation [4-HNE] and protein nitration [3-NT]) in OHCs after exposure to white noise 120 dB SPL for 1 h. Representative images and fluorescence quantification are shown in Figure 5. Indeed, the immunoreactivity of 4-HNE and 3-NT in OHCs was significantly enhanced following noise exposure. Pretreatment with 1 μM GV1001 significantly reduced the immunoreactivity of 4-HNE and 3-NT. 

## 4. Discussion

### 4.1. Summary and Interpretation of the Results

To the best of our knowledge, this is the first study to examine the hearing threshold shifts and cochlear hair cell damage rescue induced by GV1001 systemic treatment. In this study, the exposure to broadband at 120 dB SPL for 2 h resulted in significant PTS in mice; the model was useful to study the therapeutic effects of different treatments. The exposure to broadband at 120 dB SPL for 1 h induced only moderate PTS without any deterioration of OHCs but was sufficient to evaluate the levels of oxidative stress. We provide here two lines of evidence supporting the proposal that GV1001 is a promising protectant against NIHL. First, GV1001 given immediately after noise cessation rescued the hearing threshold shifts at 8, 16, and 32 kHz that developed over 2 weeks in controls and ameliorated cochlear OHC and synaptic ribbon losses. Second, GV1001 attenuated the immunoreactivities of oxidative stress, as revealed by reductions in OHC lipid peroxidation and protein nitration. Therefore, GV1001 may attenuate NIHL by reducing oxidative stress.

### 4.2. Otoprotective Effect of GV1001 on NIHL and its Mechanism Associated with Oxidative Stress

The results presented here indicate that exposure to broadband noise at 120 dB SPL for 1 h resulted in significant upregulation of 4-HNE and 3-NT expression in OHCs. The excess ROS and RNS generation is known to be associated with oxidative damage to hair cells, in a noise exposure-dependent manner [3]. Specifically, ROS and its byproducts trigger vasoconstriction and ischemia/reperfusion injury in the cochlea, namely a positive feedback loop [5]. Furthermore, the appearance of free radicals is considered an early event in the hair cell damage process triggered by exposure to high-level noise [5,19]. The free radicals were evident in hair cells before any morphological deterioration was apparent, suggesting that the radicals may play a role in damage initiation [20]. Consistent with this, we observed significantly higher levels of oxidative stress markers even 1 h after moderate PTS-noise. This finding may be based upon the notion that such noise trauma transiently deplete cellular ATP (which attain a nadir by 1 h) and activates Rho GTPases pathways that closely link to the death of OHCs in the cochlea [21].

Notably, we observed that GV1001 attenuates the levels of PTS-noise-induced oxidative stress markers in OHCs. The otoprotective effect of GV1001, as documented by reduction of hearing threshold shift and maintenance of hair cell framework against PTS-noise exposure, may be associated with the downregulation of excessive ROS and RNS expression. In accordance with this, GV1001 shows beneficial effects on kanamycin-induced ototoxicity, suggesting that its therapeutic action is associated with attenuations of oxidative stress conditions [12,13]. Considering that the ROS and RNS generation trigger inflammatory and cell stress or apoptotic pathways via ROS/RNS signaling cascades [6], GV1001 may not only directly scavenge free radical activity, but also indirectly downregulate inflammation and cell stress or apoptosis by lowering oxidative stress. GV1001 exhibits pleiotropic properties, perhaps affording protection against hearing loss caused by PTS-noise independent of ROS/RNS signaling [13]. Specifically, GV1001 has anti-inflammatory effects, inhibiting leukocyte migration and the release of pro-inflammatory cytokines such as interleukin 6 and monocyte chemoattractant protein 1 [22]. GV1001 is also neuroprotective against beta-amyloid oligomers in neural stem cells, attributable to induction of cellular proliferation, anti-apoptotic and antioxidant effects, and mitochondrial stabilization [23]. Hence, this novel peptide vaccine may have additional potential applications in other medical conditions such as sudden sensorineural hearing loss or Meniere disease on the basis of the multipotent actions on the reduction of ROS/RNS, pro-inflammatory mediators and blocking proapoptotic caspases

Interestingly, a recent study proposed that noise might loosen the blood-labyrinth-barrier; the highest perilymph concentration of the antioxidant AVN-C was noted 1 h after noise exposure [15]. In another study utilizing a guinea pig model, noise-induced ultrastructural changes in the blood-labyrinth barrier and decreased the immunoreactivities of tight junction proteins, suggesting that noise might affect blood-labyrinth barrier permeability [24]. The significant decreases in oxidative stress levels evident 1 h after PTS-noise exposure in the present study may reflect early antioxidant effects of GV1001 in the cochlea, facilitated by the pharmacodynamics of a loose blood-labyrinth barrier. GV1001, if any, may be otoprotective even when systemically administered after cessation of PTS-noise. Although the ABR test before and after treatment was not performed in the second experimental condition, our preliminary study that explores the preventive role of GV1001 against the severe PTS-noise (white noise 120 dB SPL for 2 h) has shown that the GV1001 group showed significantly fewer threshold shifts at all frequencies, compared to the saline group. It gradually improved over time and showed a threshold shift of less than 20 dB at 2-week evaluation (unpublished data) (Figure S1). Considering the hearing threshold of moderate PTS-noise in the second experimental condition, preventive administration of GV1001 would gradually lower the hearing threshold over time and result in the normal threshold. In addition, Previous studies have shown an inverse correlation between extent of oxidative stress in the cochlea and threshold shift [3,25]. That is, a significant decrease in oxidative stress levels after PTS-noise exposure may reflect early antioxidant effects of GV1001 in the cochlea. Supporting this, drugs that exert the antioxidant properties have shown to attenuate NIHL. Our findings suggest that GV1001 may serve as a preventive or post hoc treatment for acute cochlear damage induced by PTS-noise. The therapeutic timepoint window within which GV1001 restores a supposed PTS should be investigated, which in turn would have translational potential against NIHL.

### 4.3. PTS-Noise-Induced Cochlear Damage: Discrepancy between Morphology and Function

In this study, PTS-noise-induced significant loss or disorganization of OHCs, especially in the middle and basal turn. Previous studies identified that immunolabeling of byproducts of ROS and RNS persist in the cochlea 7–10 days post-noise, exhibiting gradient from the basal to the apex [26,27]. As presented here, the susceptibility of basal and middle turn of the cochlea may be attributable to a higher concentration of free radical species resulting from PTS-noise, in a concentration-dependent manner. Our finding is in line with recent studies demonstrating the correlation of noise concentration with OHC loss in the basal and middle turns of the cochlea, but not in the apical turns [3,28]. Interestingly, OHCs of apical turn of the cochlea were almost intact in all group, although there was a significant difference in threshold shift at 8 kHz under severe PTS-noise exposure, approximately 60 dB in saline group and 30 dB in GV1001 and dexamethasone groups. Specifically, a place-frequency map of the cochlea in mice was established for characteristic frequencies corresponding to the locations of basilar membrane length [29]. Previous study have shown that the mean basilar membrane length of mouse cochlea is 5.13 mm [29]. From a simple logarithmic computation based upon the normalized distance from the base and frequency, the slope of the function amounts to 1.25 mm/octave. Based on this, the 8 kHz-sensing region in the mouse cochlea lies about 1 mm from the apex of the cochlear sensory epithelium [30]. The discrepancy between the morphology and function of cochlear apical turn after severe PTS noise may be attributed to the loss of or damage to stereocilia. Previous studies suggested that PTS noise may cause the loss of stereocilia and compromise mechanical transduction before any obvious sign of damage such as fractures, folds, fusions, and/or giant hair formation [3,30]. Interestingly, ultrastructural studies of stereocilia in noise-exposed rabbits have shown that damaged OHCs usually lack stereocilia but apical turn OHCs exhibit stereocilia-to-stereocilia fusion and giant stereocilia formation [31]. Meanwhile, in the current study, a significantly decreased number of CtBP2 puncta in the apical turn was observed, regardless of treatment groups, when considering the number of vesicles tethered by a ribbon ranges from 100 to 200 in mouse cochlear IHCs [32]. Although a previous study showed that a substantial synaptic loss leads to a decrease in suprathreshold ABR amplitude, the ABR threshold shift was not apparent especially the apical turn. Similarly, our findings are inconsistent with cochlear synaptopathy in humans which exclusively demonstrate high-frequency hearing loss. Furthermore, particularly considering that OHCs are more susceptible to noise trauma than IHCs, the assumption regarding the relationship between cochlear synaptic loss and hearing threshold at apical turn is irrelevant to explain the discrepancy between the morphology and function of cochlear apical turn. 

### 4.4. GV1001 Exhibits Comparable Protection with Dexamethasone against NIHL

In the current study, dexamethasone-treated mice showed comparable protection against NIHL. In detail, GV1001-treated mice exhibited similar threshold shifts over 2 weeks with preserved OHCs and ribbon synapses, compared with dexamethasone. Previous studies have shown that noise exposure can activate the nuclear factor kappa B (NF-κB) signaling cascade, resulting in the up-regulation of the proinflammatory mediators [33]. Additionally, proinflammatory mediators may enhance cellular damage by increasing the release of reactive oxygen species (ROS)/reactive nitrogen species (RNS) from the mitochondria, leading to ROS/RNS-mediated signaling cascades which enhances programmed cell death [6]. Given the role of proinflammatory mediators in the development of NIHL, drugs with anti-inflammatory properties, such as dexamethasone, has been used for the treatment of NIHL [16,17]. In other words, the anti-inflammatory action of dexamethasone may cause the reduction of oxidative stress, which in turn may be involved in the reduction of cochlear damage induced by severe PTS noise. Indeed, in this study, a significant reduction of oxidative stress following GV1001 treatment was similarly observed in dexamethasone-treated mice (unpublished data). Meanwhile, the pleiotropic properties of GV1001 may offer additional protective effect against NIHL by reducing the upregulation of pro-inflammatory mediators and inhibiting apoptosis [22]. Thus, it appears that the comparable protective effects of GV1001 and dexamethasone against NIHL may involve the reduction of ROS/RNS generation and its signaling cascades including the pro-inflammatory mediators.

### 4.5. Strengths and Limitations of the Current Study

This is the first study to suggest that GV1001 attenuated NIHL by lowering the level of oxidative stress. Although our findings are significant, several limitations should be considered in further investigations. Our findings suggest that GV1001 may protect against NIHL by lowering oxidative stress, and may serve as preventive or adjuvant treatment, but changes in the levels of oxidative stress markers following GV1001 administration immediately after cessation of noise stimulation were not determined in this study. Thus, further studies are warranted to support the hypothesis that the protective effect of GV1001 against NIHL was due to the attenuation of oxidative stress. Our results are also limited by the fact that the experimental model only used female 4–6-week-old C57/BL6 mice. Previous several studies have shown that susceptibility and site vulnerability to noise exposure differ with strain, age, and sex [34,35]. Thus, further validation studies in male mice is needed before considering a phase I clinical trial. Additionally, the elucidation of the other damage mediators that affect ROS and RNS generation, such as intracellular free Ca^2+^ and NADPH oxidase, was not elucidated in this study; thereby, further investigation is warranted to verify whether these mediators are cooperated withGV1001 to attenuate NIHL. In detail, the ROS and RNS generation have been associated with dysregulation of Ca^2+^ homeostasis and NADPH oxidase expression, even though the precise origin and mechanism of generation of free radical species resulting from noise exposure remain elusive. Previous literature indicates that acoustic overstimulation increases the Ca^2+^ trafficking into the hair cells via intracellular and extracellular routes, subsequently changing mitochondrial membrane permeability to allow ROS release by the mitochondria [36,37]. In other words, Ca^2+^ and ROS can crosstalk between intracellular organs [38]. The dysregulation of Ca^2+^ homeostasis, in turn, may induce cytoplasmic ROS accumulation that links to hair cell damage process. Furthermore, previous studies demonstrated the reciprocal relationship of ROS and RNS overproduction with NADPH oxidase in a dose-dependent manner based on the existing notion that NADPH oxidase acts as active ROS and RNS producing enzyme that does not require stimulation or activator [39]. Therefore, we cannot exclude other potential mechanisms by GV1001 to rescue the noise exposure-induced hearing impairment. Lastly, we did not analyze the ABR amplitude between groups. Specifically, it seems meaningful to explore the amplitudes (wave I or wave III) between the GV1001 and the dexamethasone group, where the extent of hearing recovery against PTS-noise was similar. Further study is necessary to verify whether ABR amplitude correlates with the synaptic count, which would confer additional support the rescue effects of antioxidants in terms of the synaptopathy. 

## 5. Conclusions

In conclusion, GV1001 reduces hearing threshold shifts and cochlear hair cell damages induced by PTS-noise in a mouse model. Notably, GV1001 attenuates the levels of oxidative stress, including ROS and RNS byproducts in OHCs, although this study does not explore whether GV1001 directly or indirectly affects the up- and downstream pathways involved in ROS/RNS generation. However, it may suffice to say that GV1001 possibly ameliorates NIHL by lowering oxidative stress. Our results enhance our understanding of oxidative stress-mediated acute SNHL in the context of the otoprotective effects of GV1001 on NIHL.

## Supplementary Materials

The following are available online at https://www.mdpi.com/2076-3921/9/2/112/s1, Figure S1: Preliminary data that explores the preventive role of GV1001 against the severe PTS-noise.
