# Supplementary figures and images for: Outcomes of Peptide Vaccine GV1001 Treatment in a Murine Model of Acute Noise-Induced Hearing Loss
Source: Antioxidants (Basel). 2020 Jan 27;9(2):112. doi: 10.3390/antiox9020112 (PMC7070461; doi:10.3390/antiox9020112)

8 kHz

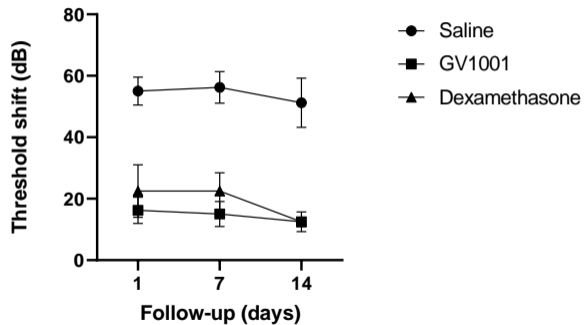

16 kHz

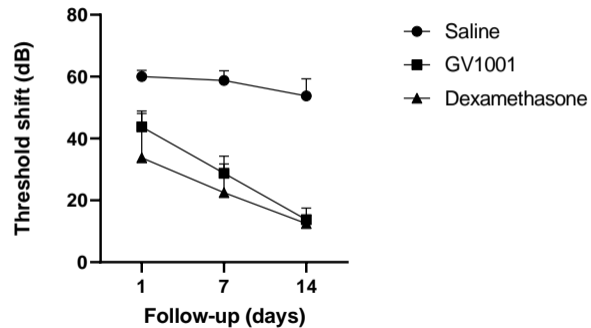

32 kHz

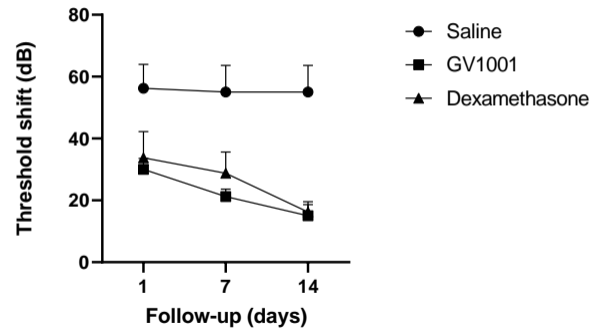

Supplement: Supplementary file 1 [file antioxidants-09-00112-s001.pdf]
